# On the Diagnosis of Abscess of the Liver

**Published:** 1884-12

**Authors:** Wm. Johnstone Fyffe

**Affiliations:** Late Assistant Professor of Medicine, Army Medical School, Netley; Physician to Clifton College


### ON THE DIAGNOSIS OF ABSCESS OF THE LIVER.

BY

WM. JOHNSTONE FYFFE, M.D.,

*Late Assistant Professor of Medicine, Army Medical School, Netley; Physician to Clifton College.*

---

ABSCESS of the Liver is not a disease of frequent occurrence in this country. It is principally to be found among residents in warm climates. Cases, however, are occasionally met with, especially among persons who have returned to England after spending some part of their lives in the East Indies or other tropical climate. Nor are those who have never left our own shores entirely exempt from attacks of hepatic abscess.

The remarks in the following paper will have reference principally to the diagnosis of liver abscess, and to some of the sources of error which may come in our way in attempting to form such diagnosis correctly.

The symptoms of this disease are often very obscure; they follow no regular course. In some instances they are unmistakeably prominent and clear; in others they are absent altogether. They often simulate diseases of adjacent organs, and frequently baffle the practitioner by assuming a mask which hides the real malady.

Rouis, in the cases collected by him in Algeria, found that the symptoms were only perfect in 8 per cent.; imperfect in 79 per cent.; and in 13 per cent. the disease ran a latent course.

Most writers of authority speak of the difficulty in certain cases of determining the presence of hepatic abscess from the almost entire absence of any symptoms to call attention to the liver. Frerichs, Budd, and Morehead record cases in which there were no hepatic symptoms, no pain or tension, diarrhœa or sickness, or any local discomfort—nothing but some amount of pyrexia; and yet such cases have proved fatal in a few days, and abscesses containing from half-a-pint to a pint of pus have been found in the liver.

Although it is not my intention to enter upon the pathological aspects of this disease, a few words are necessary as bearing upon the question of diagnosis, and especially with reference to the connexion between suppuration of the liver and dysenteric ulceration of the intestine. The almost exclusive pyæmic theory of liver abscess of some authors appears to me to be untenable; for example, 86 per cent. of the cases of hepatitis treated by Morehead recovered, and this is inconsistent with a theory of pyæmia. In Dr. Baly's cases of dysentery at the Milbank Penitentiary there was not one followed by hepatic abscess. The ulcerations of the intestine occurring in typhoid fever and phthisis are not often followed by hepatic abscess. In the Pathological Museum of the Army Medical Department at Netley there are 43 specimens of hepatic abscess; in 34 the abscess was uncomplicated with dysentery.

On the other hand, however, we have the authority of Annesley in support of the pyæmic theory. Of 29 cases collected by him, 21 had dysentery. A large proportion of Dr. Budd's cases also had a history of dysentery.

Without accepting an exclusive pyæmic theory, there is enough to show that a previous history of dysentery, especially in the cases of persons who have resided in the tropics, should have its due weight in assisting us to form a correct diagnosis.

A previous history of dysentery has also this further significance, that it will enable us to calculate with some degree of probability as to whether the suspected abscess is single or multiple, as the latter form is in the majority of cases preceded by dysenteric ulceration, although it would be unwise to lay down any hard rule on the subject.

There is another form of inflammation of the liver of occasional occurrence among tropical invalids, I mean that involving the peritoneal covering, or peri-hepatitis. That this envelope should suffer, and very frequently *does* suffer, from exudative inflammation in connexion with abscess of the parenchyma of the gland, cannot be a matter of surprise; but inflammation of this structure alone is a rare disease, and when it does occur, seldom ends in suppuration.

The detection of an abscess in the liver is not always an easy matter. I have on several occasions seen physicians who have had long practice among such cases at fault.

The introduction of exploration by puncture and aspiration into practical medicine has greatly cleared away the difficulties surrounding this special diagnosis. It was formerly thought a terrible thing to plunge an exploring needle into such a gland as the liver, and, as it were, to prospect for pus. This is an operation constantly done now, not only without risk, but with absolute benefit to the patient, even though no abscess is reached. Hepatic pain and tenderness are frequently relieved by the abstraction of a small quantity of blood in this way, and in doubtful cases it is the best mode of solving the difficulty.

The following case is, I think, a good illustration of this doctrine: A soldier, aged 41, came under my care in 1873. He was a native of Lorraine, and had formerly belonged to the German Legion. When that corps was disbanded he entered the Line, and went to India, where he served fourteen years, and enjoyed fair health until November, 1872, when he was seized with dysentery. He recovered from this, and was subsequently attacked with hepatitis, but not in an acute form; in fact, from what I could learn from his official documents, the symptoms were more those of hepatic congestion than of any inflammatory condition. When he first came under my observation he complained of severe boring pain in the right shoulder. There was fulness and prominence of the right lower intercostal spaces; the whole hepatic region was enlarged and tender; tongue slightly furred; pulse about 100; temperature *normal;* no jaundice; no fluctuation could be detected; no pointing could be observed, nor any indication that the abscess (if one existed) was near the surface. There had been no rigors, no sweating (a symptom I have observed very frequently present in hepatic abscess), and the history of the case did not give much help. The question was, Was this a case of general enlargement and congestion of the liver, or was there an abscess? Exploration appeared to be the only means of discovering the truth. On the hypothesis that if it were an abscess, the collection of matter must be large, and it seemed hazardous to wait for some further change. An exploring puncture was therefore made in the eighth intercostal space in the right side. On applying the aspirator, only about 1½ ounce of bloody fluid was removed. A second puncture farther back was made with a similar result. If there was an abscess, evidently it had not been reached. The local depletion, however, afforded considerable relief. After waiting for two days, another puncture was made a few inches still farther back. The abscess was now reached, and 12 ounces of fetid pus removed. Still, however, there was very little abatement of the pain, tenderness, and general discomfort of the patient, and no perceptible diminution of the hepatic enlargement. Accordingly a *fourth* exploration was made, and on this occasion with complete success, as I succeeded in removing no less than 96 ounces of pus by the aspirator. The fluid was measured in my presence, and the quantity was exactly six pints of 16 ounces each. I scarcely thought it possible that recovery could take place after such enormous destruction of tissue as must have occurred in this case, and yet this man recovered rapidly.

Various opinions were held among some of my colleagues as to the diagnosis in this case. Some doubted that it was an abscess of the substance of the liver, but held that it was a large collection of pus between the liver and ribs, resulting from peri-hepatitis, and circumscribed by powerful adhesions; and they based that view upon the conjecture that if the abscess had been situated in the liver itself, it must have totally destroyed the whole of the right lobe at all events, and that death would have been inevitable. Others suggested that the case was one of empyema mistaken for liver abscess, an error which has not unfrequently been made. The history of the case however, entirely excluded this view; while as regards the site of the abscess being external to the liver, resulting from suppurative inflammation of its covering, I can only say such cases are extremely rare. Morehead, whose experience in this disease is vast, considers peri-hepatitis resulting in suppuration a very unusual occurrence. Then as regards the size of the abscess, and the apparent impossibility of any of the substance of the liver being left, we must bear in mind the extreme amount of distention which the liver will bear under these circumstances, and the rapid manner in which the cavities of abscesses not lined by very thick membrane contract after evacuation. In reference to the *size* of the abscess in this case, there are many cases on record of much larger collections of matter in the liver than this. In the Pathological Museum at Netley there are several specimens of larger abscesses—one especially, which contained *seventeen* pints. But the remarkable feature in this case was the rapid and satisfactory recovery after free evacuation.

Collections of purulent matter, however, do occasionally take place external to the liver, and between the opposing peritoneal surfaces, or between the liver and diaphragm, and give rise to symptoms which are with great difficulty distinguished from those of hepatic abscess. One case is fresh in my memory of this kind, and in which the diagnosis formed at first was entirely wrong. The patient had served for some years in India, and had experienced the usual diseases to which men are generally exposed there, occasional attacks of dysentery and "liver," as it is called; but no definite symptoms of abscess until his arrival in England, when he came under my notice. There was great bulging of the right side, and distinct fluctuation between the eighth and ninth intercostal spaces in front. A puncture was made, and several ounces of pus discharged. The opening was kept free, and the discharge encouraged. Eventually several other openings formed spontaneously, and the case was treated as a liver abscess by drainage. After a long illness this patient died. There was no abscess of the liver at all; that organ was perfectly healthy; but between its external surface and the ribs there was a circumscribed abscess containing six to eight ounces of pus, and there was extensive ulceration of the structures on the inner surface of the ribs, with which the external fistulous openings communicated. From this case and some others I have met with I have been led to the opinion that when an abscess forms in this locality, between the external surface of the liver and the ribs, it generally makes its way to the surface by *several* openings; while if the abscess be seated within the liver, if it points externally, it is generally in one direction, and opens by a single orifice, the point of direction being most frequently, so far as I have observed, close to the free margin of the right lower ribs.

Abscess of the liver sometimes presents symptoms which may be mistaken for those of ulcer of the stomach, as the following case exemplifies: The patient had been a resident in India. There was no history of dysentery or (so far as we could ascertain) of liver disease. He was in a very weak, emaciated, and anæmic state. There was one spot of tenderness in the epigastrium; pressure upon this excited nausea. There was frequent and incontrollable vomiting and pain after taking food. There was no discomfort in the side, no bulging, no increase of the area of hepatic dulness, no elevation of body temperature during any part of the day. The patient died eventually, and an abscess was found in the upper and posterior part of the right lobe of the liver.

In another case of which I have the notes, the patient had returned from India in a very anæmic state. There was incontrollable vomiting. One tender spot was found on the epigastrium not larger than a half-crown piece; but manipulation of the hepatic region generally was borne without shrinking. In this case there was no history of dysentery. The diagnosis was doubtful at first. High temperature and night sweats, however, supervened, and became constant, and this cleared up the doubt. The case proved fatal. There were several abscesses in the liver; one especially was found bulging from the posterior surface, involving the œsophagus and cardiac end of the stomach. These parts were matted together in a mass.

These two cases illustrate the significance of persistent and incontrollable vomiting as a symptom of hepatic abscess. I will refer to this point farther on.

Cancer of the liver, that is, the medullary form of the disease, sometimes leads to the suspicion of abscess. I attended a physician in practice near Southampton, in consultation with Dr. Orsborn, of that town. In this gentleman's case there were some doubtful signs. There was a soft globular fluctuating tumor situated low down in the hepatic region. It was not very painful or tender. We gave the patient the benefit of the doubt, and explored the tumor. A very small quantity of turbid fluid was withdrawn. An examination of this, and the fact that there was a distinct sulcus between the upper edge of the tumor and the free margin of the false ribs, led us to the conclusion that the case was one of malignant growth from the lower edge of the liver, which view was proved to be correct at the *post-mortem* examination.

Distention of the gall-bladder and echinoccoci passing into suppuration, may each give rise to the suspicion of hepatic abscess; but the diagnosis in such cases is easy in comparison to those cases where there exists pleuro-pneumonia at the base of the right lung, accompanying suppurative inflammation of the liver. In such cases there is, however, a degree of prostration which no amount of stimulating food seems to alleviate, and which leads to the suspicion that there is a graver complication than merely inflammation of the lower lobe of the right lung and pleura; and, of course, the previous history either of dysentery or tropical residence will aid the diagnosis.

Abscess of the lung may be mistaken for hepatic abscess, as shown in the following case: A soldier, aged 38, was attacked with severe hepatic pain. The organ was apparently much enlarged. He had cough, but no expectoration. Shortly after admission he was seized with exacerbation of pain apparently in the liver, and expectorated a large quantity of pus, streaked with blood and very offensive. The case appeared to be one of hepatic abscess bursting through the right lung, and was treated as such. Gradually the expectoration declined and the health improved, when a second time there was a sudden aggravation of the above symptoms, with signs of breaking up in the lower lobe of right lung. The patient eventually died. The right lung was found compressed in its upper part; a large abscess was found in the lower and middle lobes, the lower boundary in intimate connexion with the diaphragm and convex surface of the liver, the former being very thin and reduced to the consistence of tissue paper. There was no abscess in the liver, or any trace of former inflammation in that organ.

The following case is an illustration of an almost directly opposite condition of things; namely, the existence of an hepatic abscess bursting through the right lung, and afterwards mistaken for an advanced stage of phthisis. A man, aged 25, was invalided from India for fever. On the passage home he had several attacks of ague (so called, but doubtless they were rigors indicating the formation of abscess); he also had suffered from acute dysentery. On his arrival in England he was found to he suffering from cough, purulent expectoration, and night sweats. The physical examination indicated a disorganised condition of the right lung from top to bottom. The case was regarded as one of rapid phthisis. The expectoration was sanguino-purulent at first, afterwards purulent. Death took place from exhaustion and hectic. At the *post-mortem* examination the left lung was found healthy; the lower and middle lobes of the right were found infiltrated and broken up by purulent deposits, the lower lobe being attached to the diaphragm, through which an enormous abscess of the liver, containing nearly three pints, had burst.

It may be said that the history of the case, the condition of the right apex, and the character of the sputa should have directed the diagnosis; but when cases of this kind are seen, as they most frequently are in this country, only in an advanced stage, the diagnosis is difficult. The dark prune-juice expectoration, characteristic of liver-abscess, has often ceased, and it has become simply purulent. Microscopic examination of the sputa may help us, but it is not always to be relied on.

The symptoms of hepatic abscess may be complicated with others of so formidable a character as to throw doubt and uncertainty over the whole diagnosis. The following case is an evidence of this fact; the case presents some unusual clinical and pathological features: J. H., aged 29, a man of temperate and regular habits; served for five years in India. During his service there he suffered from ague on three occasions in 1871-72. He never had hepatic disease or dysentery in India.

From 1873 until 1875 he continued in good health, when he received a gunshot wound of the hand, which necessitated his being invalided, and he arrived at Netley on the 2nd February, 1876. The wound was healed. He was admitted to the medical wards for apparently a feverish cold. On the 4th he was pale; his breathing and pulse both accelerated. After careful examination a small prominence was noticed over the left lobe of the liver, about two inches below the sternum, and in the mesial line. It was rather hard on pressure, but neither tender or painful. Pressure caused a slight feeling of nausea. There were no rigors or perspirations. Next day the tumor had increased in size, and on that evening the temperature rose to 104, fell next morning to 100, and fluctuated between that point and 102 until the 7th, when it suddenly fell to 97, above which point it never rose again. The tumor increased in size, became more defined, with a boggy sensation, and with a distinct sense of *pulsation* at the sides. The patient had several attacks of syncope. On the 11th, at 8.45, he passed about 1½ pint of blood by the rectum. The temperature sank to 96·4. There continued to be occasional vomiting, faintings, and very rapid pulse until the 13th, when the temperature sank to 95. He died on the 14th, twelve days after his admission to Netley Hospital.

At the *post-mortem* examination the following conditions were found: There was an abscess, as at first supposed, in the left lobe of the liver, its walls without any distinct lining membrane, its contents very thick and purulent. The liver was pushed forward by the transverse colon, which projected below it; and this, together with the whole of the large intestine, was distended with blood and quite black. The small intestine was throughout normal except in calibre, being in its lower part reduced to the diameter of a lead pencil. In the caput coecum, but not elsewhere, there were *three* ulcers, each of the size of a sixpence, with deep bases and prominent ragged surfaces. Evidently the hæmorrhage had taken place from these, and had gradually filled and distended the large intestine with partially coagulated blood. Its passage from the anus had been partly prevented by a constriction near the orifice, the remains of old ulceration.

This case appears to me to present many points of interest; but space will not permit me to do more than to remark that the diagnosis was complicated and obscured by the insidious onset and subsequent rapid progress of the symptoms, by the position of the tumor, by its pulsation, and by the unusual appearance of hæmorrhage, syncope, and depression of temperature.

Collections of fluid in the right pleura may be, and frequently are, mistaken for abscess of the liver. I have seen several instances of this kind during the last ten years; in one case notably, where the pleuritic effusion had formed a pouch behind the diaphragm, and had pushed the liver forward very prominently. It is always best with obscure signs of this kind to make an exploratory puncture.

What, then, are the signs and symptoms most to be relied on in forming a diagnosis of hepatic abscess? In answering this question I would exclude those cases of acute hepatitis resulting in abscess, rarely seen out of the tropics. In such cases the diagnosis is comparatively easy. The pain, high temperature, rapid pulse, hepatic tenderness, frequent vomiting, and successive rigors, all point to the formation of abscess. This condition of things is rarely met with in Indian residents returned to England. The acute symptoms are seldom observed. If they have existed they have passed away. The abscess, if there be one, may have already formed, and all symptoms of inflammatory action may have ceased.

In those cases which present a great similarity to fever of an intermittent type, careful observations of temperature may be of material assistance.

When hepatic abscess is accompanied by frequent rigors the temperature rarely falls to the normal standard during the intermission, as it does in ague. The fever paroxysms are also of shorter duration, are more irregular in their rhythm, and are seldom influenced by the administration of quinine.

Hepatic pain, although present in the majority, at least, of acute cases, is not a constant symptom in those of a more chronic character, nor is it very severe.* The liver is not a very sensitive organ; and, unless the coverings of the organ be involved, the pain experienced by the patient is seldom commensurate with the amount of local mischief. Tenderness on pressure is, however, a sign which I have hardly ever observed to be absent in hepatic abscess; but the amount of tenderness depends mainly upon the position in which the abscess is situated. The cases in which tenderness is most marked, are those where the abscess is situated on the concave surface of the gland.

Increase of the area of hepatic dulness is an important sign in diagnosis of liver abscess, but we must look for it in the upper margin. The lower margin generally remains within its normal limits, unless the abscess be of very large size. Auscultation, I need not say, is of great importance in enabling us to determine the relation between the lung and upper margin of the liver.

* The sensation is one more of discomfort and fulness rather than fixed pain.

Enlargement of the liver, by which I mean general swelling of the organ in all directions, is not of frequent occurrence in suppurative inflammation. This fact has been specially noted by Morehead, who explains it upon the hypothesis that the nutrient vessels, namely, the capillaries of the hepatic artery, are those most engaged in inflammation; that their capacity is much smaller than those of the portal vein; and that this in a great measure accounts for the fact that the bulk of the organ is little increased in inflammation compared with congestion, a deranged condition in which the large portal capillaries are directly implicated. And certainly this view, if correct, tends to explain another fact, that the function of secretion is seldom interfered with in cases either of acute or chronic suppurative inflammation of the liver. I do not remember ever to have seen jaundice occurring in the course of an hepatic abscess. Out of 318 cases Morehead only observed jaundice in *five;* its presence or absence is, therefore, of no account in determining the diagnosis. There is, however, a dusky condition of the skin, especially of the face, neck, and upper part of the trunk, almost constantly present in these cases. Whether it be the result of the circulation of bile in the tissues, or from pigmentation following long exposure to malarial influences, I am not prepared to say.

Incontrollable vomiting occurring in a patient with a tropical history is a symptom possessing considerable diagnostic value. This has been pointed out forcibly by my late colleague in the Army Medical School, Professor Maclean, in an article published in the *British Medical Journal* in August, 1874. Dr. Maclean there cites several cases showing the significance of this symptom.

Sir Joseph Fayrer, the present physician to the Council of India, in commenting on Dr. Maclean's paper, draws attention to the insidious manner in which the liver passes into a condition of suppuration, not only in the cases of persons residing in India, but also in those who have left the country, and refers to the point, which has been borne out by his experience, that vomiting, as a symptom of liver-abscess, will depend much upon the position and size of the abscess and upon the pressure it may exercise on adjacent organs, or to the amount of irritation it may excite in the vagus nerve. The two cases I have already cited bear out this observation.

There are some cases of hepatic abscess of insidious origin, however, where there are no symptoms, and where the first indication that an abscess has existed may be the evacuation of a large quantity of purulent matter by the bowels.

I would say, in conclusion, that if in the cases of persons formerly resident in India who have returned to this country, either with or without any history of previous hepatic or intestinal derangement, we should find symptoms of malaise, more or less pyrexia, evening chills followed by perspiration not controllable by quinine, a malarial aspect, dingy conjunctivæ, vomiting difficult to subdue, with irregularity of the bowels, we may have grave suspicions of hepatic abscess; and if, in addition to these, there should be pain and discomfort in the region of the liver, sympathetic pain in the right shoulder, cough, dyspnœa, tenderness on pressure, tension of the right rectus muscle, and any prominence of the right side, our suspicion may be reduced almost to certainty. In such circumstances an exploratory puncture removes all doubt.

